# Supplementary material for: Genome-Wide Identification and Characterization of Long Non-Coding RNAs in Embryo Muscle of Chicken
Source: Animals (Basel). 2022 May 16;12(10):1274. doi: 10.3390/ani12101274 (PMC9137640; doi:10.3390/ani12101274)
Supplement: Supplementary file 1 [file animals-12-01274-s001.zip › animals-1628289-supplementary.pdf]

Supplementary material

Tabel S1. Primer sequencing in this study.

**lncRNA**	**Forward primer(5’-3’)**	**Reverse primer(3’-5’)**
XR_001471472.2	CCGGGCCTGCACTCTTGATA	GCAAATCCACACGGCCTGAC
XR_003077657.1	GATAAGGCGGTAACAACTC	ATAGGAAGCCACTCACAAA
XR_212291.3	CTCTCCTGCTGAGCAAGCCATTG	TCCCTGGATGAAGACCCGTTCTG
XR_001466777.2	TGGCATAATGGCTGAACAGGGAAG	GCACTACTCTGGGCAACCTTGG
XR_001466502.2	GGTAGGAAGATATAGGAGAGCATGC	CTTGTGGGATAGAGAGATTCACTGG
MSTRG.17928.1	GAGCTGGAATTCACTGGTAGATACC	GGGAGGAGGAGCAAATCTATAGAAG

Figure legends

Figure S1 Screening of LncRNAs. (A) The distribution of reads length for lncRNAs; (B) The exons length distribution of lncRNs and mRNAs.

Figure S2 Sample relationship analysis. (A) The PCA of lncRNA. (B) The Pearson correlation heat map between lncRNA.

Figure S3 Weighted gene co-expression network analysis (A) The Power value curve; (B) Module gene correlation analysis.

Figure S4 (A) The Functional enrichment analysis of the cis-regulation lncRNAs. A. the top 20 significantly changed biological process terms of mRNAs; B Top 20 significantly changed pathways associated with mRNAs.

Figure S1

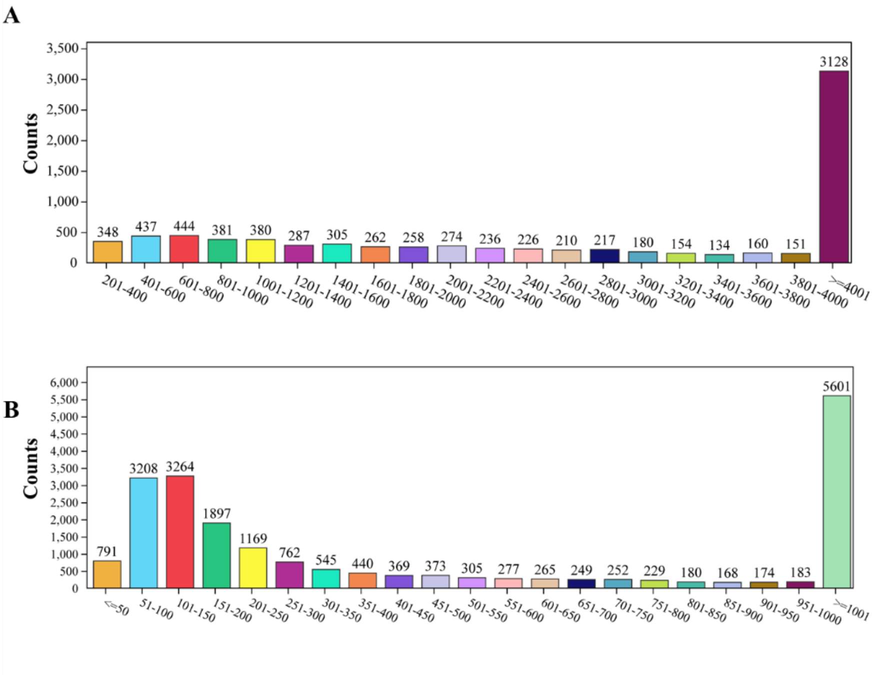

A
Counts
3,500
3,000
2,500
2,000
1,500
1,000
500
0
348
437
444
381
380
287
305
262
258
274
236
226
210
217
180
154
134
160
151
3128
201-400
401-600
601-800
801-1000
1001-1200
1201-1400
1401-1600
1601-1800
1801-2000
2001-2200
2201-2400
2401-2600
2601-2800
2801-3000
3001-3200
3201-3400
3401-3600
3601-3800
3801-4000
>=4001
B
Counts
6,000
5,500
5,000
4,500
4,000
3,500
3,000
2,500
2,000
1,500
1,000
500
0
791
3208
3264
1897
1169
762
545
440
369
373
305
277
265
249
252
229
180
168
174
183
5601
<=50
51-100
101-150
151-200
201-250
251-300
301-350
351-400
401-450
451-500
501-550
551-600
601-650
651-700
701-750
751-800
801-850
851-900
901-950
951-1000
>=1001

Figure S2

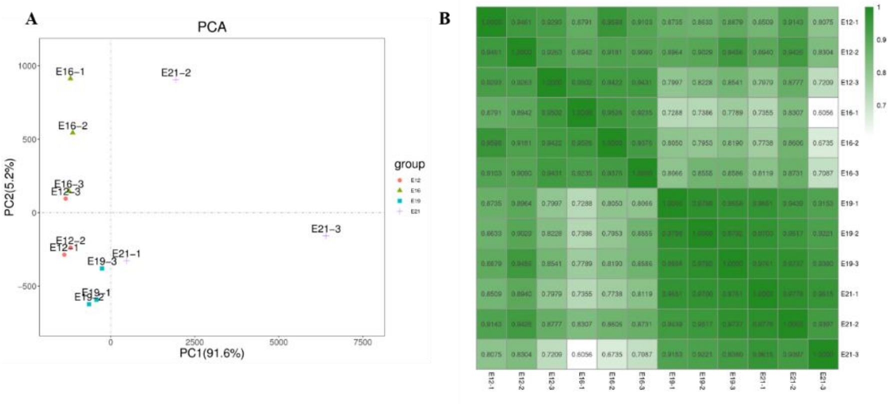
A
PCA
E16-1
E21-2
E16-2
E16-3
E12-3
E12-2
E12-1
E21-1
E19-3
E19-1
E19-2
E21-3
group
E12
E16
E19
E21
PC2(5.2%)
PC1(91.6%)
1000
500
0
-500
0
2500
5000
7500
B
E12-1
E12-2
E12-3
E16-1
E16-2
E16-3
E19-1
E19-2
E19-3
E21-1
E21-2
E21-3
1
0.9
0.8
0.7

Figure S3

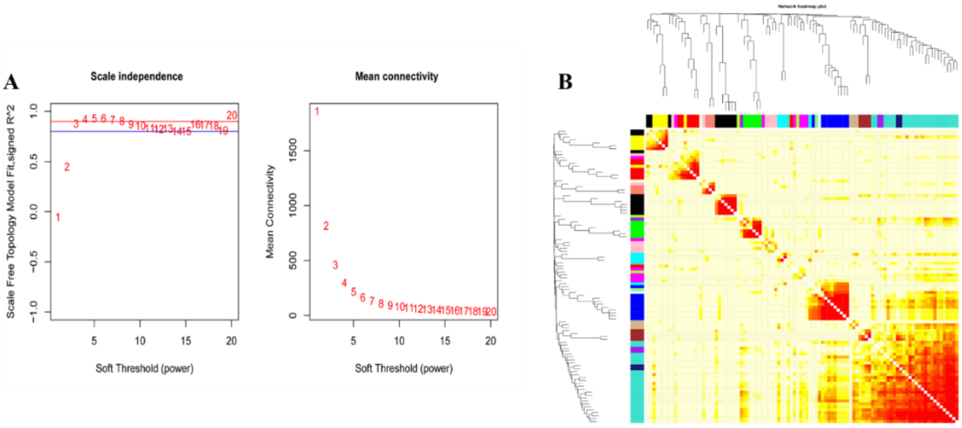
A
Scale independence
Scale Free Topology Model Fit,signed R^2
Soft Threshold (power)
Mean connectivity
Mean Connectivity
Soft Threshold (power)
B

Figure S4

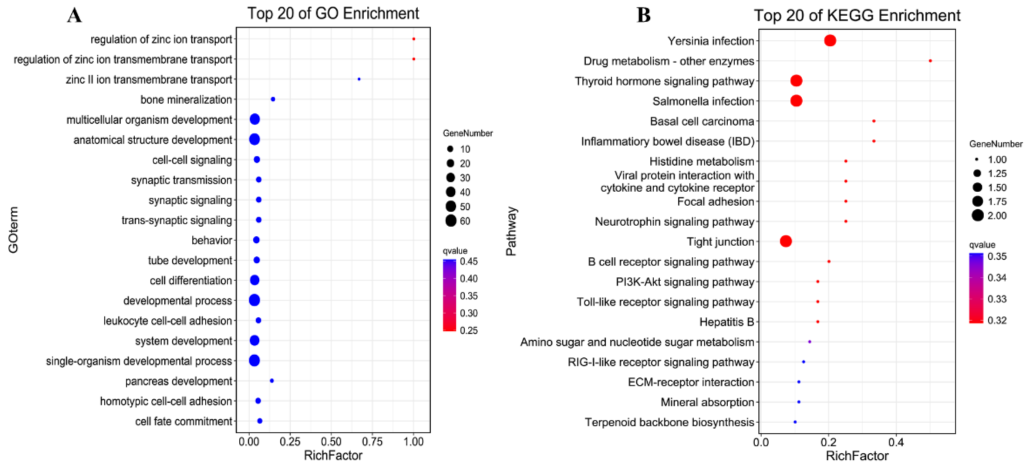
A
Top 20 of GO Enrichment
regulation of zinc ion transport
regulation of zinc ion transmembrane transport
zinc II ion transmembrane transport
bone mineralization
multicellular organism development
anatomical structure development
cell-cell signaling
synaptic transmission
synaptic signaling
trans-synaptic signaling
behavior
tube development
cell differentiation
developmental process
leukocyte cell-cell adhesion
system development
single-organism developmental process
pancreas development
homotypic cell-cell adhesion
cell fate commitment
GOterm
0.00 0.25 0.50 0.75 1.00
RichFactor
GeneNumber
10 20 30 40 50 60
qvalue
0.45 0.40 0.35 0.30 0.25
B
Top 20 of KEGG Enrichment
Yersinia infection
Drug metabolism - other enzymes
Thyroid hormone signaling pathway
Salmonella infection
Basal cell carcinoma
Inflammatory bowel disease (IBD)
Histidine metabolism
Viral protein interaction with cytokine and cytokine receptor
Focal adhesion
Neurotrophin signaling pathway
Tight junction
B cell receptor signaling pathway
PI3K-Akt signaling pathway
Toll-like receptor signaling pathway
Hepatitis B
Amino sugar and nucleotide sugar metabolism
RIG-I-like receptor signaling pathway
ECM-receptor interaction
Mineral absorption
Terpenoid backbone biosynthesis
Pathway
0.0 0.2 0.4
RichFactor
GeneNumber
1.00 1.25 1.50 1.75 2.00
qvalue
0.35 0.34 0.33 0.32
